# Documentation Displaces Teaching in an Academic Emergency Department

**DOI:** 10.5811/westjem.2020.5.46962

**Published:** 2020-06-15

**Authors:** Joshua J. Baugh, Derek L. Monette, James K. Takayesu, Ali S. Raja, Brian J. Yun

**Affiliations:** Harvard Medical School, Massachusetts General Hospital, Department of Emergency Medicine, Boston, Massachusetts

## Abstract

**Introduction:**

Adverse effects of administrative burden on emergency physicians have been described previously, but the impact of electronic health record documentation by academic emergency attendings on resident education is not known. In this observational study of a quaternary care, academic emergency department, we sought to assess whether the amount of time attending physicians spent on documentation affected the amount of time they spent teaching.

**Methods:**

A fourth-year emergency medicine (EM) resident observed 10 attending physicians over 42 hours during 11 shifts, recording their activities every 30 seconds. Activity categories were developed iteratively by the study team and validated through co-observation by an EM education fellow with a kappa of 0.89. We used regression analysis to assess the relationship between time spent documenting and time spent teaching, as well as the relationship between these two activities and all other attending activity categories.

**Results:**

Results demonstrate that time spent documenting was significantly and specifically associated with less time spent teaching, controlling for patient arrivals per hour; every minute spent on documentation was associated with 0.48 fewer minutes spent teaching (p<0.05). Further, documentation time was not strongly associated with time spent on any other activity including patient care, nor did any other activity significantly predict teaching time.

**Conclusion:**

Findings suggest that academic attendings may face a trade-off between their documentation and teaching duties. Further study is needed to explore how administrative expectations placed on academic emergency physicians might interfere with trainee education.

## INTRODUCTION

Academic emergency physicians are asked to balance a myriad of demands during shifts. In addition to providing clinical care and completing administrative tasks, they must find time to teach trainees. Prior studies suggest that time spent teaching correlates with perceived teaching quality by trainees,^1^ and that expert academic physicians find creative ways to incorporate education into clinical shifts.^2^ Previous studies from over a decade ago found that clinical work load did not significantly affect teaching quality during shifts.^3^,^4^ However, with increasing administrative tasks and the introduction of (EHR) health records, it may be that modern demands on academic physicians do interfere with teaching opportunities.

Documentation burden in particular has been shown to affect many facets of physician life;^5^–^7^ it may follow that documentation impacts education in the clinical setting as well. However, there is no study to our knowledge that has examined the direct impact of documentation on teaching in an emergency medicine (EM) setting. In this observational study, we therefore sought to assess how time spent on documentation activities affected time spent on teaching by attending physicians in one academic emergency department (ED). We also examined whether documentation time was associated with time spent on direct patient care or other attending activities, and whether activities other than documentation impacted time spent teaching.

## METHODS

This study was conducted in an urban, quaternary care, academic center with an EM residency that receives approximately 110,000 patient visits per year. It was considered quality improvement by the institutional review board and therefore exempt from review. All observations were conducted in the 25-bed critical care area of the ED, which sees 52 patients per day of whom 60% are admitted. This area is supervised by one attending at all times of day, with varying levels of staffing by residents and physician assistants depending on time of day. In general, all patients in our ED are seen by a resident or physician assistant; only very rarely do attendings see patients on their own.

A fourth-year EM resident observed attendings during shifts, writing down all activities they performed in 30-second intervals. Forty-two total clinical hours were observed in 10 four-hour blocks and one 2-hour block, all between the hours of 10 am and 7 pm. Specific shifts to be observed were chosen at random without regard to who the covering attending would be. One attending was observed in each period, and 10 different attendings were observed over the course of 11 observation blocks (one attending was observed twice by chance). Attendings were not aware of the aims of the study and were therefore unlikely to have systematically altered any particular behaviors in response to being observed.

Attending activity categories were developed iteratively by the study team and then validated through co-observation by a second observer (an ED education fellow) over two separate two-hour co-observation sessions. During these sessions, each observer simultaneously and independently used the iteratively designed rater scale to record and code attending activities in 30-second intervals. Subsequently, observations were compared by 30-second increment for agreement.

Teaching categories included bedside teaching, procedural teaching, case-based teaching, didactic teaching, and implicit teaching through case discussion, all explicitly defined and then identified by the observers. Time spent on any teaching category was then summed to create a total teaching time variable. Documentation time consisted of all time a physician spent creating a patient care note, which included typing, dictating using computer software, or dictating to a scribe. This was separate from “chart review,” which consisted of attendings reviewing results data or previous medical history on the computer. Direct patient care consisted of all time attendings spent in patient rooms.

The primary outcome was the relationship between teaching, documentation, and patient care during shifts. This was assessed using univariate regression analyses between these variables and multivariate regression controlling for patient arrivals per hour. In the area where observations were performed, attendings must see all patients upon arrival; attendings do not have discretion over which patients they see. Therefore, arrivals per hour captured the number of patients that attendings saw, and attendings could not modulate their patient load based on other factors. Secondary outcomes involved further regression analyses to assess the association of other attending activities with teaching time and documentation time, respectively. Finally, a sensitivity analysis with removal of outliers assessed the potential impact of outliers on results.

## RESULTS

### Co-observation

The two observers had a Cohen’s kappa of 0.89. Of note, they achieved 90% agreement on teaching categories specifically.

### General Characteristics

On average, attendings in our study spent 32% (standard deviation [SD] 9%) of their time on direct patient care, 25% (SD 7%) on teaching, and 12% (SD 8%) on documentation. They spent 7% (SD 6%) of time on socializing and breaks, 6% (SD 2%) on chart review, 6% (SD 2%) receiving sign-out, and the remaining time on other activities including taking emergency medical services calls, communicating with non-trainee team members, walking, and speaking with consultants. Attendings saw a median of 2.9 (SD 0.59) patients per hour and spent a median of 5.8 (SD 2.6) minutes in each patient’s room.

### Outcomes

Increased time spent on documentation was associated with significantly decreased time spent teaching in our sample (Figure). In a univariate regression model, every additional minute of documentation time predicted 0.48 fewer minutes of teaching during a shift (p = 0.04). This relationship was not affected by controlling for the number of patient arrivals per hour (Table), suggesting patient volumes did not explain the inverse relationship between time spent documenting and teaching. In further univariate analyses, patient care time was not significantly associated with teaching time (coefficient 0.12, p = 0.6), nor was time spent on chart review (coefficient 1.5, p = 0.13), breaks and socializing (coefficient −0.21, p = 0.57), or any other observed activity. Documentation time was also not significantly associated with time spent on direct patient care (coefficient 0.13, p = 0.66) or any activity other than teaching.

Through sensitivity analysis, we identified and removed one outlier (involving an unusually high amount of teaching for our sample). Removal of this outlier strengthened the findings; the direction and magnitude of the coefficient between teaching and documentation was unaffected, but the p value on this coefficient decreased from 0.04 to 0.0007. The relationship remained robust to controls for all other activities. This suggests outliers did not drive our main outcome, and, in fact, weakened it.

## DISCUSSION

Our results suggest that time spent on documentation by attendings was specifically associated with decreased time spent on teaching in this academic ED, while time spent on direct patient care, chart review, breaks, and other activities was not significantly associated with teaching time. This may indicate attendings face a particular trade-off between documentation and teaching demands. A recent study found that introducing residents into a community ED setting did not slow attending productivity because increased time spent teaching and supervising was balanced by the completion of administrative tasks by residents; this suggested a trade-off between education time and administrative time (one assumes these community physicians spent only as much time teaching as the decrease in administrative tasks allowed, since productivity was unaffected).^8^ Our results fit with this pattern; when attendings spent less time on documentation they spent more time teaching, and vice versa. As academic departments weigh documentation expectations for faculty, they may wish to consider potential educational effects.

Of course, we do not know whether increased documentation caused less teaching or was simply associated with less teaching; it may be that documenting directly interferes with teaching or that attendings who devote more time to documentation simply tend to teach less. While the latter remains possible, documentation may plausibly impact teaching at multiple junctures. If attendings are documenting during or immediately after patient presentations by trainees, they may not seek opportunities to probe or teach. An attending with a moment of free time may face a choice between gathering trainees to discuss a case and finishing notes. An attending documenting in a patient room may more likely miss out on opportunities for bedside teaching.

In our department, attendings write their own patient care notes. Recent EHR data from our department suggests attendings spend an average of more than one hour working on the medical record after shifts. The 12% of shift time we observed attendings spend documenting, therefore, likely underestimates total time spent on documentation. It may be that attendings who spent more time teaching pushed their documentation duties until later; if so, attendings who taught more paid a price with more uncompensated time spent documenting after shifts.

## LIMITATIONS

Our study was small and occurred in a single academic department with particular documentation requirements for attendings. This may limit the generalizability of our findings. Our sample size did not allow for analysis by attending characteristics, such as tenure. More research is certainly needed to assess whether patterns observed persist with larger samples across institutions. We were also unable to assess perceptions of teaching quality and whether increased time spent teaching was associated with better teaching. While prior research has suggested an association between time spent teaching and teaching quality, only time spent teaching could be captured in our study. In addition, we were unable to assess time spent documenting after shifts were finished. We were therefore only able to capture time spent documenting during shifts, rather than total time spent on documentation by attendings.

## CONCLUSION

Our study suggests that documentation demands placed on attending emergency physicians may be associated with less time for teaching in academic departments. Further work should examine how this may affect teaching quality, as well as the amount of off-shift administrative time required of attendings. It will be important to better understand how administrative burden on attendings impacts resident education, and what can be done to optimize ED educational environments in the face of administrative responsibilities.

## Figures and Tables

**Figure:**
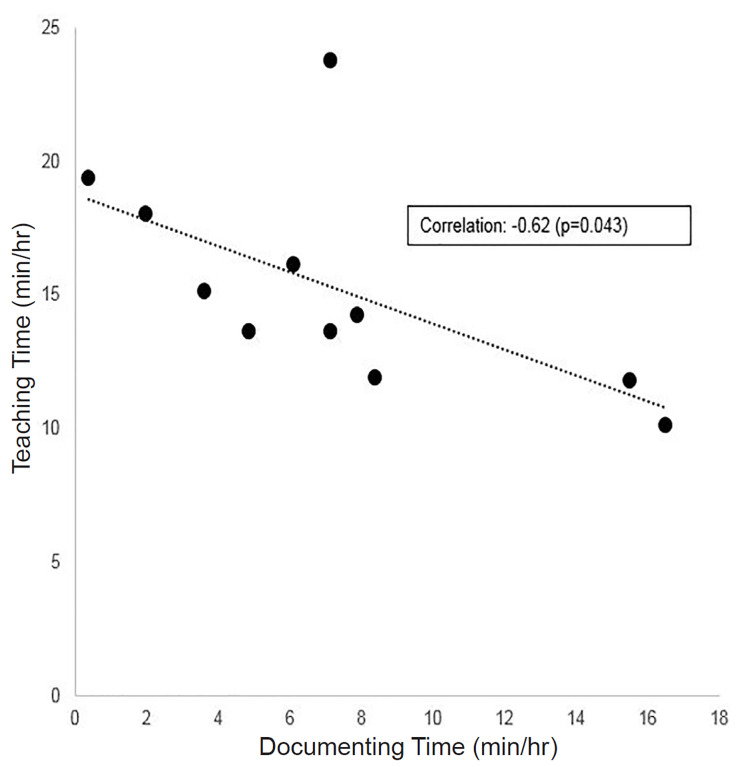
Relationship between time spent teaching versus documenting during an hour of on-shift time (n = 11). *Min*, minute; *hr*, hour.

**Table:** Univariate regression analysis results for the associations of time spent teaching with time spent documenting, and patient arrivals per hour with time spent teaching, along with a multivariate regression of the association of time spent teaching with time spent documenting, controlling for patient arrivals per hour.

Relationship of interest	Coefficient (Standard Error)
Association of minutes spent documenting with minutes spent teaching	−0.48 (0.18)**
Association of patients per hour with minutes spent teaching	−0.006 (0.56)
Association of documenting with teaching, controlling for patients per hour	−0.5 (0.21)**

** p<0.05
